# Use of direct oral anticoagulants for chronic thromboembolic pulmonary hypertension

**DOI:** 10.6061/clinics/2018/e216

**Published:** 2018-05-08

**Authors:** Francisca Alexandra Gavilanes-Oleas, Jose Leonidas Alves, Caio Julio Cesar Fernandes, Luis Felipe Lopes Prada, William Salibe, Mario Terra, Luciana Morinaga, Susana Hoette, Carlos Jardim, Rogerio Souza

**Affiliations:** Unidade de Circulacao Pulmonar, Instituto do Coracao (InCor), Hospital das Clinicas HCFMUSP, Faculdade de Medicina, Universidade de Sao Paulo, Sao Paulo, SP, BR

**Keywords:** Pulmonary Thrombosis, Pulmonary Hypertension, Direct Oral Anticoagulants

## Abstract

**OBJECTIVES:**

Chronic thromboembolic pulmonary hypertension is one of the most prevalent forms of pulmonary hypertension and is a major complication of acute pulmonary embolism. One mainstay of chronic thromboembolic pulmonary hypertension treatment is lifelong anticoagulation. The recent advent of direct oral anticoagulants for acute pulmonary embolism treatment has provided a viable and effective alternative for treating this condition. However, little is known about the efficacy of this new class of drugs for treating chronic thromboembolic pulmonary hypertension. We aimed to evaluate the safety and efficacy of direct oral anticoagulants in the treatment of chronic thromboembolic pulmonary hypertension.

**METHODS:**

A cohort of chronic thromboembolic pulmonary hypertension patients who initiated treatment with direct oral anticoagulants between June 2015 and November 2016 were enrolled in this study.

**RESULTS:**

Sixteen patients used rivaroxaban, three used dabigatran and one used apixaban for a mean follow-up of 20.9 months. The mean age was 51 years, and eighteen patients were classified as functional class II/III. Eight patients underwent a pulmonary endarterectomy and exhibited clinical, hemodynamic and functional improvement and currently continue to use direct oral anticoagulants. No episode of venous thromboembolism recurrence was identified during the follow-up period, but there was one episode of major bleeding after a traumatic fall.

**CONCLUSIONS:**

Although direct oral anticoagulants appear to be a safe and effective alternative for treating chronic thromboembolic pulmonary hypertension, larger studies are needed to support their routine use.

## INTRODUCTION

Acute pulmonary embolism (PE) is a highly prevalent condition, and with up to 3 million associated deaths per year, it is the third most common cause of cardiovascular death in the world 1. However, currently, systemic anticoagulation is an effective therapy for acute PE. Since the 60s, several studies have demonstrated the impact of intravenous or subcutaneous anticoagulation on mortality of acute PE patients and the capacity of anticoagulation for preventing recurrence 2,3.

Oral vitamin K antagonists (VKA) were also successfully evaluated for use in anticoagulation therapy for acute PE 4. Nevertheless, the pharmacological properties of VKA pose several challenges for their long-term use, which led researchers to pursue newer, safer and better oral anticoagulation alternatives for treating general and, in particular, acute PE. Direct oral anticoagulants (DOACs) (dabigatran, rivaroxaban, apixaban and edoxaban) have recently been shown to be an effective and a possibly safer alternative to conventional anticoagulation agents 5 in the treatment of acute PE. These new drugs are now the first choice for treating acute PE unrelated to malignancy 6. Nevertheless, the efficacy and safety of DOACs have not been studied in one of the most severe complications of acute PE, chronic thromboembolic pulmonary hypertension (CTEPH).

CTEPH is a serious, potentially fatal disease and one of the most prevalent forms of pulmonary hypertension (PH), defined by the presence of mean pulmonary artery pressure ≥ 25 mmHg 7,8. Data suggest that 0.1 to 8.8 % of acute PE patients may develop CTEPH 9. Known risk factors for the chronicity of PE are chronic inflammatory and infectious diseases, high recurrence rates of venous thromboembolism (VTE) and genetic predisposition 7.

One of the mainstays of CTEPH therapy is lifelong anticoagulation, for which VKA are routinely recommended 10. However, whether the benefits of DOACs that were identified in acute PE patients are also present in the CTEPH population has yet to be demonstrated.

We report a cohort of 20 CTEPH patients treated with DOACs between June 2015 and November 2016.

### Case Series Report

In this study, sixteen patients used rivaroxaban, three patients used dabigatran, and one patient used apixaban. Demographic and functional data are shown in Table 1. The median time of DOAC use was 20.9 months.

In eight patients, DOACs were used before and after pulmonary endarterectomy (PEA; also known as pulmonary thromboendarterectomy or PTE), which is the main therapeutic intervention for CTEPH 11. However, the remaining 12 patients had contraindications for the surgical procedure or were on a waiting list.

No episode of VTE recurrence was identified during the follow-up period. Concerning safety, there was one episode of major bleeding after a traumatic fall that resulted in the death of the patient. Another patient died during the immediate postoperative period of PEA, but there was no association with the use of DOAC.

## DISCUSSION

In the present study, DOACs were successfully used in CTEPH patients without any evidence of VTE recurrence and with low rates of major bleeding, suggesting that DOACs are a secure and effective option for anticoagulation therapy for CTEPH.

CTEPH is a progressive disease believed to be related to inadequate dissolution of an acute thrombus, followed by its fibrotic organization. A fundamental step in CTEPH treatment is lifelong anticoagulation, and thus, current guidelines recommend the use of VKA for this purpose. Nevertheless, the long-term use of VKA is particularly challenging due to the pharmacological properties of these drugs, such as narrow therapeutic range, several drug and food interactions and the continuous need for monitoring. DOACs have demonstrated a number of advantages over the use of VKA in several conditions. Clinical trials recently demonstrated that these new drugs are at least as effective as and possibly safer than VKA in managing acute lung thrombosis 12. Nevertheless, data regarding the safety and efficacy of DOACs in the CTEPH population are still lacking.

Bleeding is the most feared adverse effect of any anticoagulation treatment. In this case series, one patient presented significant bleeding related to trauma that resulted in death, but no recurrence of PE was identified during the follow-up period. Of note, the use of DOACs did not interfere with surgery or the postoperative follow-up period.

This study is a single-center case series with all the associated intrinsic limitations of such an investigation. Patients were neither randomized nor VKA-controlled. Nevertheless, our results are promising and suggest that DOACs are a safe and effective alternative therapy for lifelong anticoagulation of CTEPH patients, thus emphasizing the need for prospective, randomized, multicenter trials to establish the routine use of DOACs in CTEPH patients.

## AUTHOR CONTRIBUTIONS

Gavilanes-Oleas FA, Alves-Jr JL and Fernandes CJ have collected data and contributed with the concept and design of the study. Prada LF, Morinaga L, Salibe-Filho W and Terra-Filho M have collected data and contributed with critical writing and revising the intellectual content of the manuscript. Hoette S, Jardim C and Souza R have contributed with analysis and interpretation of data, and final approval of the manuscript.

## Figures and Tables

**Table 1:** Patient demographic and clinical features

**Gender (M:F)**	8:12
**Age (years)**	51±16
**BMI**	28±4
**Functional class**	
**I**	
**II**	10
**III**	8
**IV**	2
**Hemodynamic data**	
**mPAP (mmHg)**	53±10
**PAOP (mmHg)**	14±5
**CO (L/min)**	3.7±1.2
**PVR (WU)**	11±4
**BNP (pg/ml)**	265±219
**6MWT (m)**	359±118
**DOAC**	
**Apixaban**	1
**Dabigatran**	3
**Rivaroxaban**	16
**Duration of use (month)**	20±14

PEA: pulmonary endarterectomy, M: male, F: female, cm: centimeters, BMI: body mass index, mPAP: mean pulmonary arterial pressure, PAOP: pulmonary artery occlusion pressure, CO: cardiac output, PVR: pulmonary vascular resistance, BNP: B-type natriuretic peptide, 6MWT: six-minute walking test, DOAC: direct oral anticoagulant.
